# Supplementary material for: Highly efficient broadband terahertz generation from ultrashort laser filamentation in liquids
Source: Nat Commun. 2017 Oct 30;8:1184. doi: 10.1038/s41467-017-01382-x (PMC5662695; doi:10.1038/s41467-017-01382-x)
Supplement: Supplementary file 1 — Supplementary Information [file 41467_2017_1382_MOESM1_ESM.pdf]

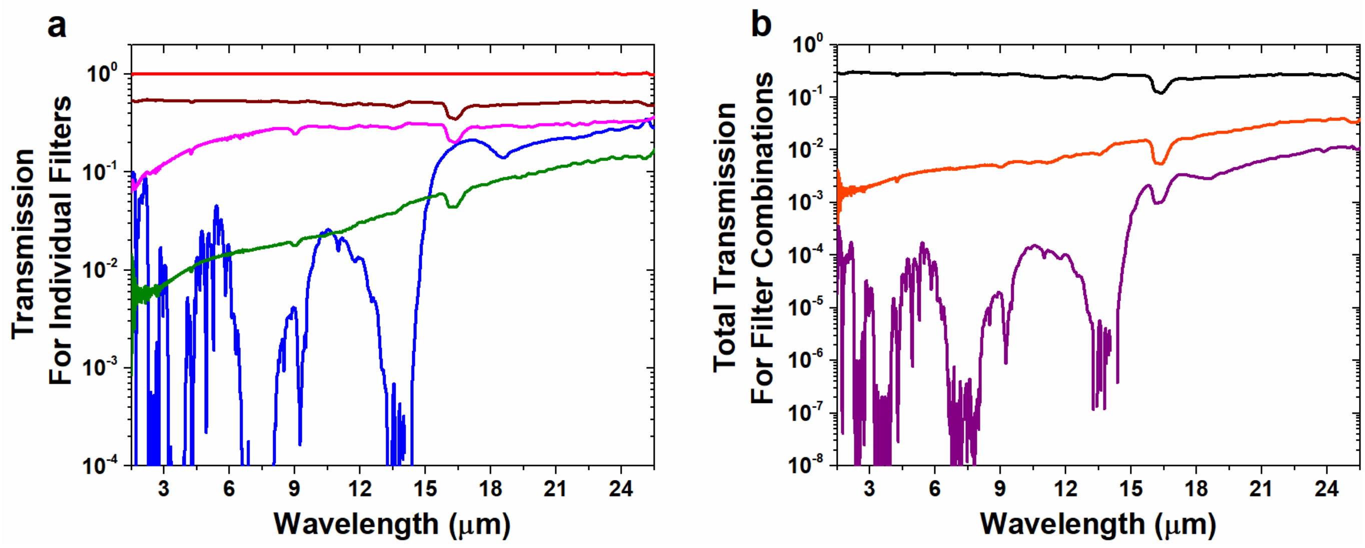

Supplementary Figure 1 | Transmission characteristics of terahertz filters used in the experiment. **a**, The filter transmission characteristics are obtained using Fourier transform infrared spectrometer (JASCO FT/IR-4100A). The characteristics are plotted for, 0.5 mm thick high-resistivity float-zone silicon (HRFZ-Si) (dark-red line), 0.5 mm thick low-resistivity silicon (LR-Si) (magenta line), black silicon on 0.5 mm thick LR-Si (Black-LR-Si) (green line), 5 mm thick high-density poly-ethylene (HDPE) (blue line). The reference transmission in absence of any filter (only air) is shown by red line. **b**, The resultant total transmission for the filter combinations: 2 × HRFZ-Si (0.5 mm) (black line), 2 × HRFZ-Si (0.5 mm) + Black-LR-Si (0.5 mm) (orange line), and 2 × HRFZ-Si (0.5 mm) + Black-LR-Si (0.5 mm) + HDPE (5 mm) (purple line) is shown. The combination 2 × HRFZ-Si (0.5 mm) + Black-LR-Si (0.5 mm) + HDPE (5 mm) is primarily used in the experiments.

Supplementary Table 1 | Average mid-infrared transmission for the various filter combinations. The integrated signal from filamentation in acetone recorded by the pyroelectric detector with the filter combinations, 2 × high-resistivity float-zone silicon (HRFZ-Si) (0.5 mm), 2 × HRFZ-Si (0.5 mm) + black silicon on low-resistivity silicon (Black-LR-Si) (0.5 mm), and 2 × HRFZ-Si (0.5 mm) + Black-LR-Si (0.5 mm) + high density poly-ethylene (HDPE) (5 mm), before and after the correction by filter transmission. The data demonstrates that the detected signal extends well beyond the mid-infrared (mid-IR) frequencies, into the terahertz region.

**Filter**	**Avg. Transmission in the mid-IR**	**Measured signal (V)**	**Filter Corrected Signal (V)**
2 × HRFZ-Si (0.5 mm)	0.3	7.58	25.2
2 × HRFZ-Si (0.5 mm) + Black-Si (0.5 mm)	5×10^{-3}	0.23	46.0
2 × HRFZ-Si (0.5 mm) + Black-Si (0.5 mm) + HDPE (5mm)	10^{-4}	0.10	1000

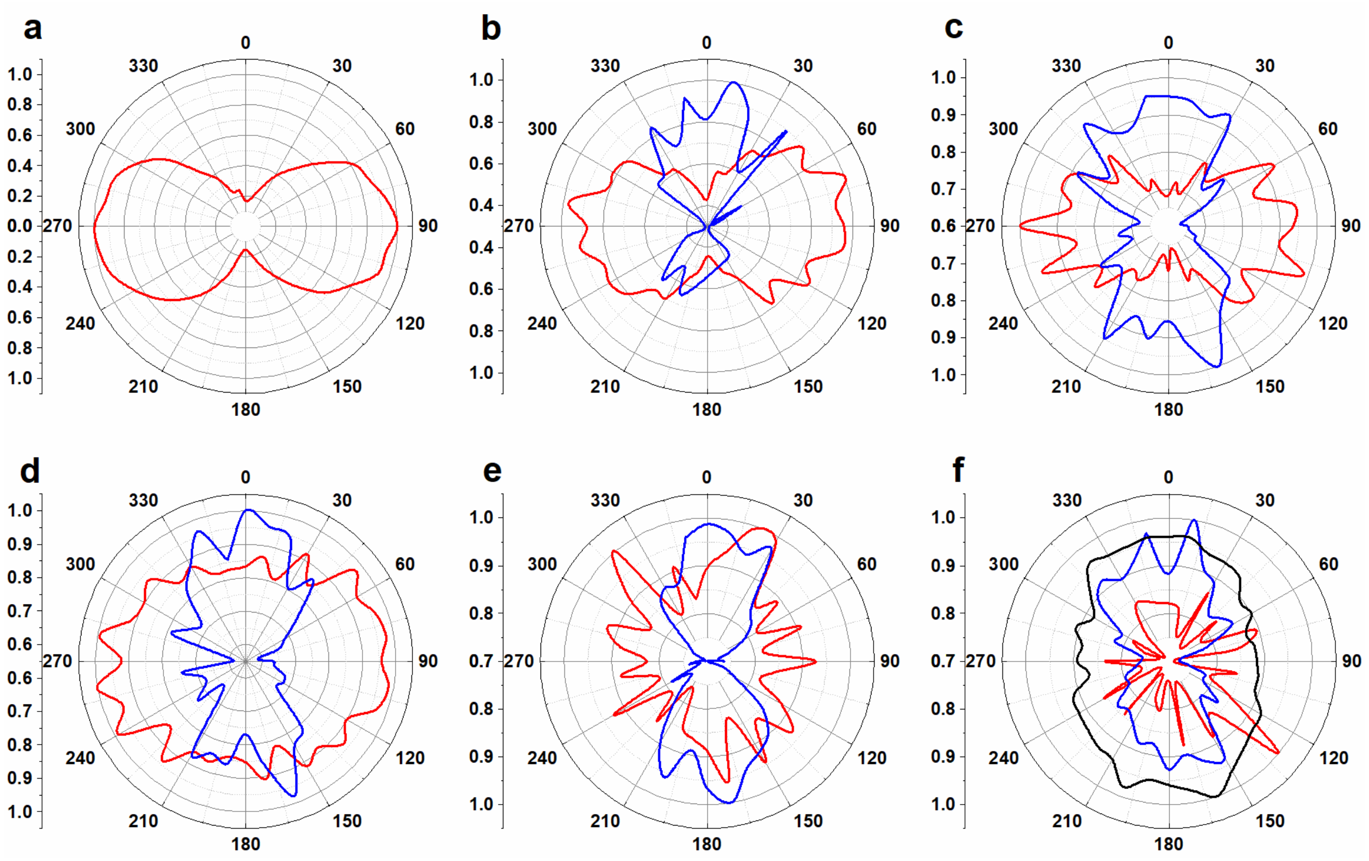

Supplementary Figure 2 | Optical polarization measurements of the 800 nm fundamental and 400 nm second harmonic from the generated supercontinuum. **a**, Normalized optical intensity plot of the polarization of the input 800 nm pulse before filamentation. Polarization plot of the 800 nm (red line) and 400 nm (blue line) after filamentation **b**, in air **c**, inside empty cuvette (air); and in acetone at incident energies (U_{L}) of **d**, 1 mJ, **e**, 6 mJ, and **f**, 10 mJ. The terahertz polarization from filamentation in acetone at 10 mJ is also shown in **f**, for reference. Note that no second harmonic crystal (BBO) is used in these measurements.

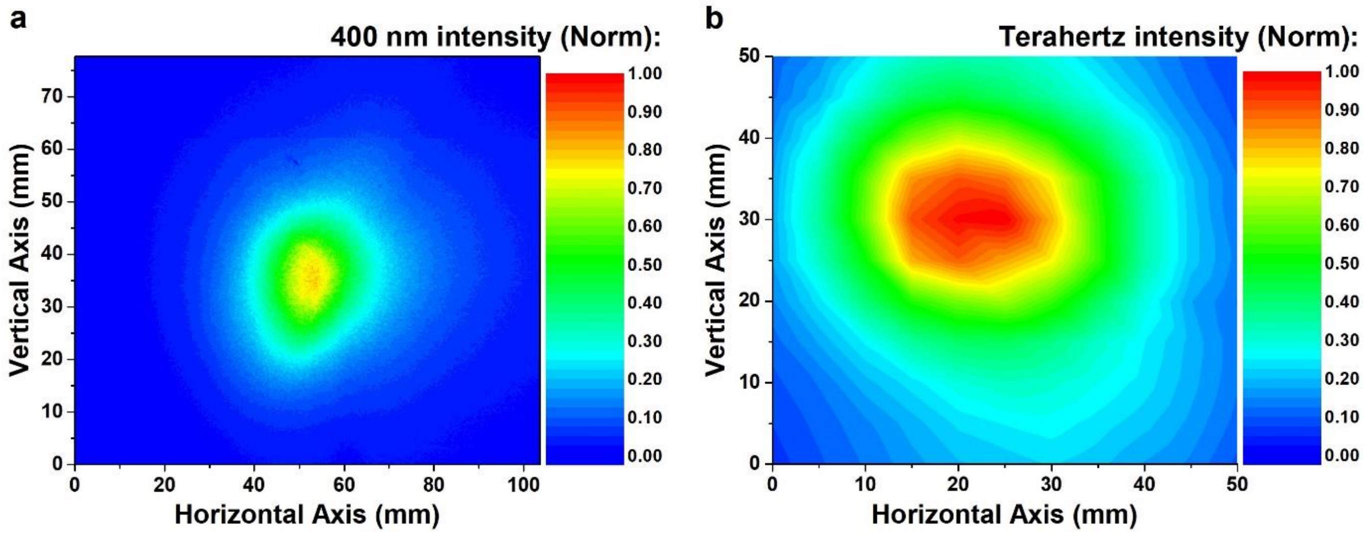

Supplementary Figure 3 | Integrated spatial profile measurements for optical and terahertz emissions. Spatial intensity profile of **a**, 400 nm emission (measured by CMOS camera), and **b**, integrated terahertz emission (measured by raster scan of pyroelectric detector), from filamentation in acetone in 5 cm cuvette at a laser energy of 10 mJ.
